# Dinitrogen Activation Mediated by the (P_2_P^Ph^)Fe Complex: Electronic Structure, Dimerization Mechanism, and Magnetic Coupling

**DOI:** 10.1021/acs.inorgchem.3c03813

**Published:** 2024-01-09

**Authors:** Jhon Zapata-Rivera, Carmen J. Calzado

**Affiliations:** † Facultad de Ciencias Naturales y Exactas, Departamento de Química, Universidad del Valle, Calle 13 N° 100–00, 25360 Cali, Colombia; ‡ Departamento de Química Física, Universidad de Sevilla, c/Profesor García González, s/n, 41012 Sevilla, Spain

## Abstract

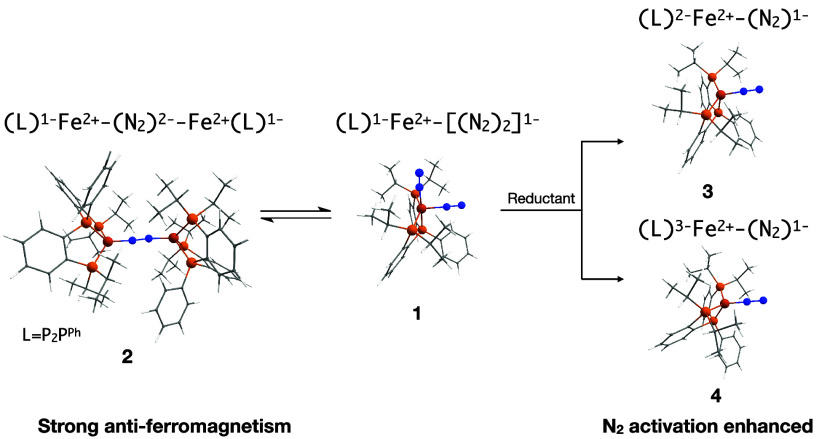

Herein, we report the estimation of the extent of dinitrogen activation by different charged and structural forms of (P_2_P^Ph^)Fe biomimetic catalysts, which, in the presence of light, exhibit significant yield in the N_2_-to-NH_3_ conversion. Complete active space self-consistent field (CASSCF) calculations have been used to determine the electronic structure of different reduced forms of the mononuclear complexes: the neutral (P_2_P^Ph^)Fe(N_2_)_2_ adduct and the anionic [(P_2_P^Ph^)Fe(N_2_)]^−^ and [(P_2_P^Ph^)Fe(N_2_)]^2–^ complexes. These calculations also revealed that the extent of reduction of a dinitrogen molecule reaches up to one electron (N_2_^1–^) due to the back-bonding from the Fe center, in agreement with the changes observed in the vibration frequency of the N–N bond in these complexes. In addition, the energy profile of the dimerization of the mononuclear (P_2_P^Ph^)Fe(N_2_)_2_ complex to the dinuclear mono-N_2_-bridged [(P_2_P^Ph^)Fe]_2_(μ–N_2_) complex has been determined by means of density functional theory (DFT) calculations. A three-step mechanism has been proposed for the dimerization, favored by both kinetics and thermodynamics criteria. Finally, the magnetic coupling constant in the diiron (μ–N_2_) complex is estimated from CASSCF/NEVPT2 calculations. Such a dinuclear complex presents a strong antiferromagnetic coupling resulting from the interaction between two *S* = 1 d^6^ Fe^2+^ ions, bridged by a highly activated dinitrogen molecule (N_2_^2–^) with two electrons on the π* orbitals.

## Abstract 2

The electronic structure analysis of different (P_2_P^Ph^)Fe(N_2_)_*x*_ catalysts revealed the enhancement of N_2_ activation, reaching a reduction to N_2_^1−^, due to the back-bonding from the Fe atom. The extent of dinitrogen activation changes with nuclearity and reduction of the complex.

## Introduction

**Scheme 1:**
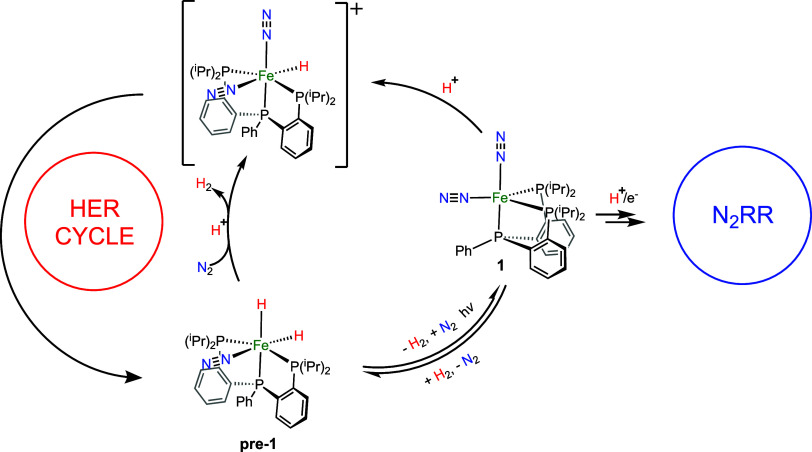
Structures of Catalytic Species Connecting HER and N2RR Cycles

The interest in the photocatalytic conversion of dinitrogen to ammonia has been revived as an alternative to the traditional Haber–Bosch process.^1−3^ Because of the natural abundance of N_2_ in the air and the possibility of using the light as a source to its catalytic activation, photosynthesis of ammonia from dinitrogen is seen as one of the most promising and sustainable alternatives to the Haber–Bosch process.^4−6^ Several studies have shown the formation of ammonia and nitrates from the nitrogen reduction reaction (N_2_RR) using a variety of semiconductor catalysts and biomimetic systems in the presence of light.^7−9^ Biomimetic photocatalysts capable of coordinating and reducing N_2_ are often built on the basis of an iron center, emulating the active site of the nitrogenase reductase enzyme.^10−14^ Fe catalysts have low efficiency toward the N_2_RR because of the competing hydrogen evolution reaction (HER). This reaction produces dihydrogen by a proton-coupled electron transfer process. Therefore, striving to have higher ammonia yields using bioinspired compounds, the catalysts proposed must have greater selectivity for N_2_ molecules than for H_2_. In this vein, Peters et al.^15^ have synthesized and characterized several hydrides of biomimetic Fe complexes with phenylphosphine ligands. Specially, the dinuclear hydride [(P_2_P^Ph^)Fe(H)]_2_(μ–N_2_) (one hydride per Fe atom) and the mononuclear dihydride (P_2_P^Ph^)Fe(N_2_)(H)_2_ (**pre-1**; Scheme 1) showed comparable ammonia yielding.^16,17^ In the dark, these photocatalysts exhibit low yield in the N_2_-to-NH_3_ conversion. However, when these complexes are illuminated in the ultraviolet–visible (UV–vis) region, the yield of ammonia increases significantly owing to the formation of the (P_2_P^Ph^)Fe(N_2_)_2_ (**1**) complex, which is on-path for the N_2_RR via a proton-coupled electron transfer reaction (Scheme 1). This enhancement in the yield of ammonia makes **1** an ideal model to the in-depth study of the N_2_ activation.

**Scheme 2:**
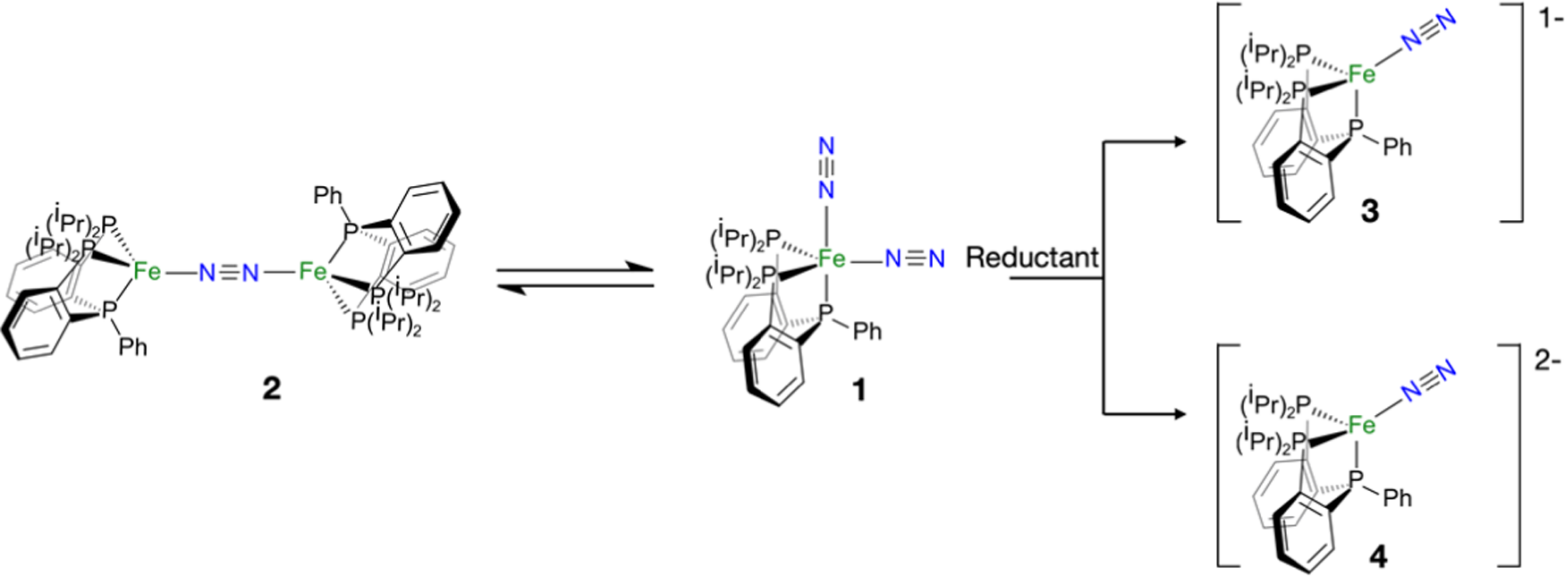
Structures of Species Resulting from Dimerization (Left) and Reduction (Right) of **1**

Furthermore, as depicted in Scheme 2, the same authors have also reported an equilibrium between **1** and the diiron mono-N_2_-bridged complex [(P_2_P^Ph^)Fe]_2_(μ–N_2_) (**2**) in solution. Complex **2** behaves as a molecular antiferromagnet, as emerged from ^1^H and ^31^P NMR data; however, no mechanistic studies about the formation of **2** from the dimerization of **1** have been reported. In addition, the reduction of **1**, coupled to the release of one N_2_ molecule, results in the formation of a salt of anions [(P_2_P^Ph^)Fe(N_2_)]^−^ (**3**) or/and [(P_2_P^Ph^)Fe(N_2_)]^2–^ (**4**), depending on the equivalents of reductant agent used (Scheme 2). **3** and **4** are found to be more active species for the N_2_RR than **1**(17) because the N_2_ ligand is sufficiently activated to produce ammonia by its protonation in acid medium. In fact, the N–N vibration frequency is reduced to 1872 and 1677 cm^–1^ for **3** and **4**, respectively, compared to the value of the isolated molecule (2358.6 cm^–1^). Such anionic species are four-coordinated complexes with doublet and singlet ground states, respectively, in which the oxidation state of the Fe center and the extent of the N_2_ activation were not unambiguously assigned.

Some efforts have been made in the description of the stability and electronic structure of Fe–N_2_ type complexes by means of density functional theory (DFT)-based techniques.^18−20^ These DFT approaches have proven reliable performance in the estimation of geometries and reaction mechanisms. However, the intrinsic multiconfigurational character of this kind of systems advocates the use of wave function theory (WFT)-based methods to describe the electronic structure and charge transfer processes.

We then envisaged a two-part study devoted to the characterization of these complexes and the photocatalytic processes involving them. In this first part, we mainly focus on the determination and analysis of the electronic structure of complexes **1**–**4**, while work is in progress to characterize the photochemical transformations of (P_2_P^Ph^)Fe catalysts. We present here the electronic structure characterization of complexes **1**–**4** by means of complete active space self-consistent field (CASSCF) calculations. Such a characterization allows us to estimate the changes in the oxidation state of the iron atom and the degree of N_2_ activation, which is the critical process for the nitrogen reduction reaction to take place. The NEVPT2 approach has also been used to evaluate the magnetic coupling constant of complex **2**. We have also taken advantage of the DFT approach to determine the dimerization mechanism of complex **1** to give complex **2**. This study hopefully brings insights into the key interactions governing N_2_ activation by Fe-based biomimetic catalysts.

## Results and Discussion

### Electronic Structure of Mononuclear Species

**Scheme 3:**
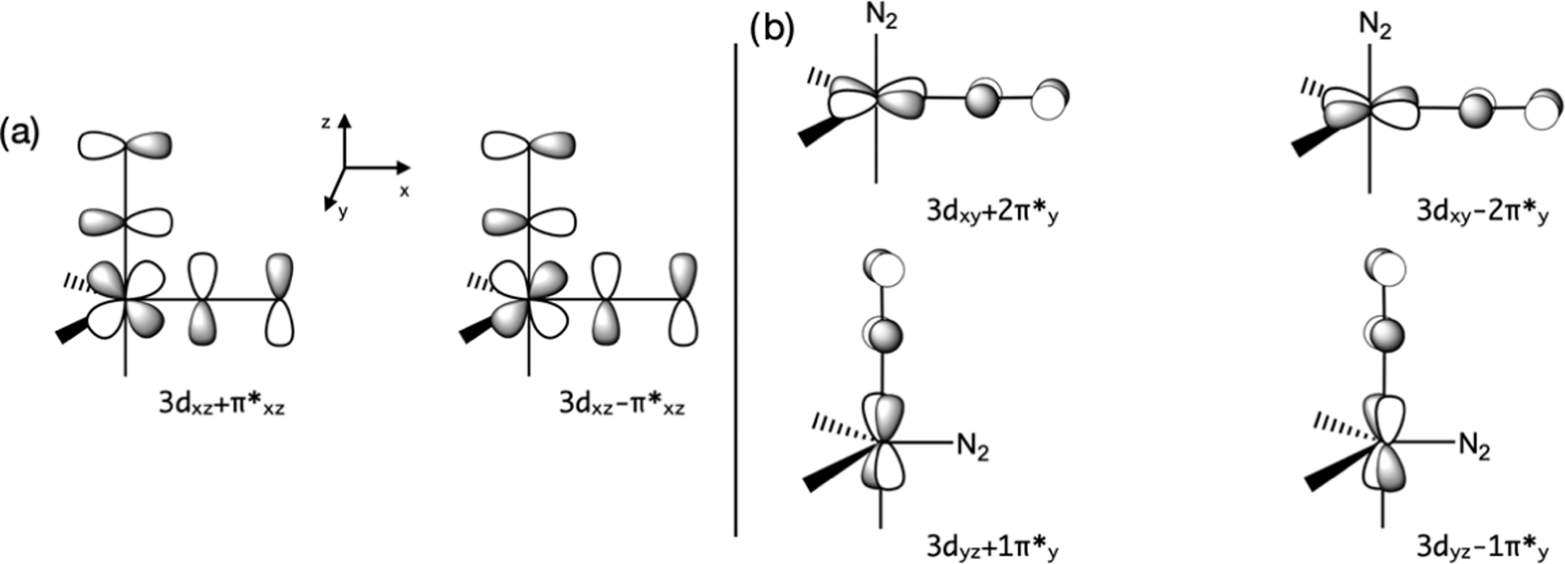
Schematic Representation of the d–π Interaction between the Fe and N_2_ Fragments

We have analyzed the electronic structure of mononuclear species (**1**, **3**, and **4)** by means of CASSCF calculations. In complex **1**, the Fe center presents a distorted trigonal bipyramidal coordination, where the N_2_ molecules occupy the axial and one of the equatorial positions (Scheme 2). From test calculations, we found that in this coordination, the Fe 3d_*xz*_ orbital interacts with the combination of the π* orbitals (π*_x_ and π*_z_) of the N_2_ molecules (π*_xz_ in Scheme 3a).

**Figure 1:**
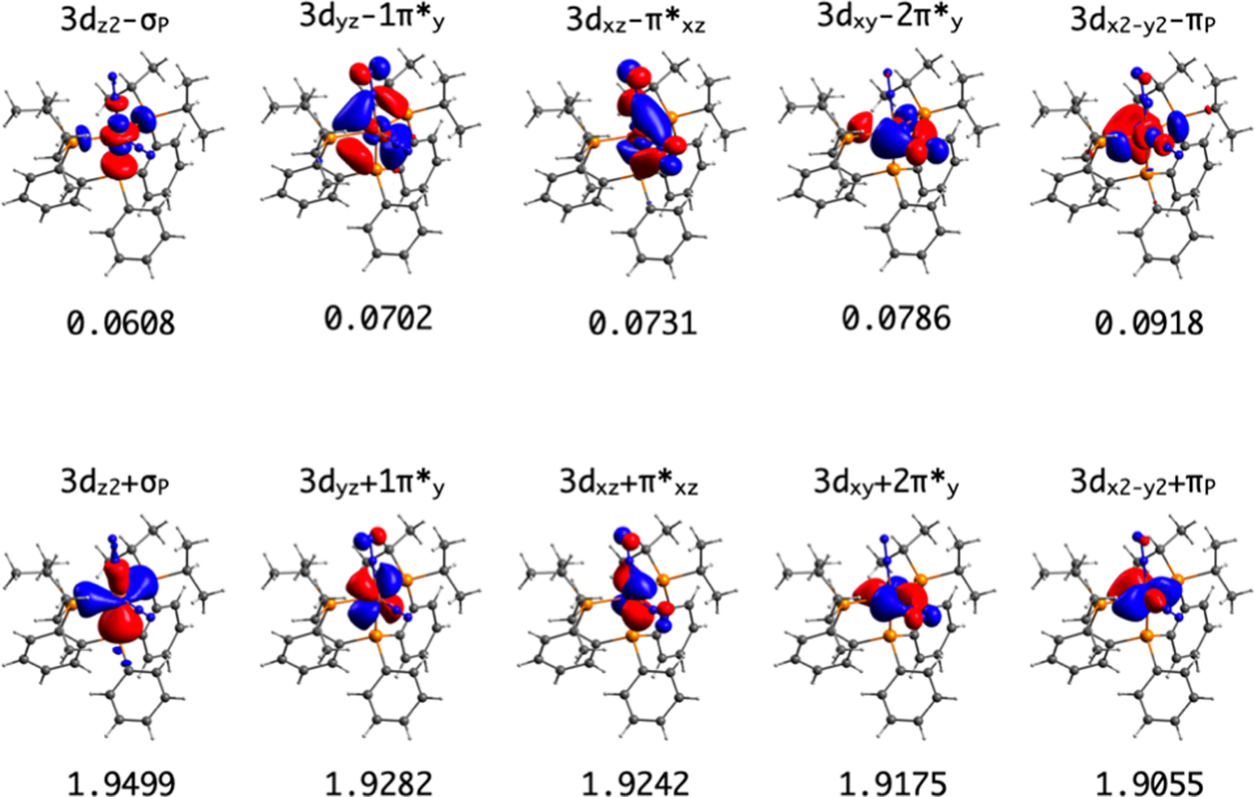
Active MOs of the singlet ground state of complex **1**. Occupations are included.

In addition, the Fe 3d_*xy*_ and 3d_*yz*_ orbitals strongly overlap with the π*_y_ orbital of each N_2_ molecule (Scheme 3b). Furthermore, Fe 3d_*z*^2^_ and 3d_*x*^2^–*y*^2^_ orbitals, higher in energy, combine with one σ_P_ occupied and one π_P_ unoccupied orbitals of the P_2_P^Ph^ ligand, respectively. Thus, assuming eight valence electrons for the Fe atom in complex **1**, an adequate description of the electronic structure demands at least 10 electrons (8 from the Fe atom and 2 from the P_2_P^Ph^ ligand) and the quoted 10 orbitals in the active space of the CASSCF calculations (Figure 1). At the CASSCF(10,10) level, complex **1** has a singlet ground state (*S* = 0), which is stabilized by 57.1 kcal·mol^–1^ with respect to the triplet state (*S* = 1). The CASSCF wave function of the singlet state reveals that the dominant contribution is |(3d_*z*^2^_ + σ_P_)^2^(3d_*yz*_ + 1π*_y_)^2^(3d_*xz*_ + π*_xz_)^2^(3d_*xy*_ + 2π*_y_)^2^(3d_*x*^2^–*y*^2^_ + π_P_)^2^⟩, with 84% of the weight.

The analysis of the ground state wave function enabled us to establish partial populations on the P_2_P^Ph^, Fe, and N_2_ moieties and therefore to estimate their corresponding oxidation states and the extension of the N_2_ activation (see the Computational Methods Section). The active natural orbitals (NOs) of the singlet ground state reveal a high degree of covalency in the Fe–N and Fe–P bonds (Figures 1 and S4, in the Supporting Information (SI) file, contains additional figures of the active orbitals with a smaller isosurface value to show the contribution of ligand atoms). In fact, in terms of the fragment contributions to each active NO, we number 6.4 active electrons on the Fe atom, 1.1 electrons distributed on the π* orbitals of the N_2_ molecules (about 0.5 on each), and 2.5 electrons on the P_2_P^Ph^ ligand, which corresponds to a net gain of 0.5 electrons, due to a certain degree of charge transfer from the metal center (Table 1). Notice that the back-bonding does not introduce spin density on the fragments. The population of the Fe center agrees with the d^6^ configuration. Therefore, the oxidation states of the different fragments composing **1** can be roughly understood as (P_2_P^Ph^)^1–^Fe^2+^–[(N_2_)_2_]^1^ to highlight the back-bonding to the P_2_P^Ph^ ligand and, in particular, to the dinitrogen molecules, such that each N_2_ molecule becomes slightly activated. It is worth mentioning that **1** has been previously suggested as a (P_2_P^Ph^)Fe^0^–(N_2_)_2_^0^ complex.^17^ However, the experimental N–N frequencies (*v̅*_symm_ = 2065 cm^–1^, *v̅*_asymm_ = 2005 cm^–1^) are significantly lower than the value reported for the isolated N_2_ molecule (*v̅*_N–N_ = 2358.6 cm^–1^).^17,21^ This indicates that the N_2_ ligands are slightly reduced, in line with our analysis of the ground state wave function. A similar electronic distribution is displayed by the low-lying triplet state, in which the dominant contribution (85% of the weight) results from a single excitation from the (3d_*x*^2^–*y*^2^_ + π_P_) orbital to the (3d_*z*^2^_ – σ_P_) orbital.

**Table 1:** Normalized Contribution (NC) of the P_2_P^Ph^, Fe, and N_2_ Fragments to the Active MOs of the Singlet Ground State for Complex **1**^a^

	active MO occupations	1.95	1.93	1.92	1.92	1.91	0.09	0.08	0.07	0.07	0.06	Occ per fragment
NC	Fe	0.18	0.83	0.82	0.78	0.60	0.51	0.61	0.61	0.63	0.65	
	1N_2_	0.01	0.00	0.05	0.10	0.08	0.14	0.16	0.11	0.00	0.01	
	2N_2_	0.10	0.06	0.07	0.01	0.06	0.05	0.02	0.15	0.14	0.04	
	P_2_P^Ph^	0.71	0.11	0.06	0.12	0.26	0.30	0.21	0.13	0.23	0.30	
Occ	Fe	0.35	1.61	1.58	1.49	1.15	0.05	0.05	0.04	0.04	0.04	6.40
	1N_2_	0.02	0.00	0.10	0.19	0.16	0.01	0.01	0.01	0.00	0.00	0.49
	2N_2_	0.20	0.12	0.13	0.01	0.10	0.00	0.00	0.01	0.01	0.00	0.59
	P_2_P^Ph^	1.39	0.20	0.11	0.23	0.50	0.03	0.02	0.01	0.02	0.02	2.51

a The corresponding fractional occupation (Occ) per fragment is also included.

We have also simulated the UV–vis spectrum of **1** at the CASSCF(10,10) level and compared it with the experimental one. Thus, the low-lying singlet excited states of **1** have been determined and the wavelengths associated with the corresponding electronic excitations from the ground state have been evaluated (Figure S1). As shown in Table 2, the calculated wavelengths that agree with those observed in the UV–vis spectrum (490, 405, and 322 nm)^17^ are at 497, 430/418, and 328 nm, respectively, which are associated with the populations of the first, second/third, and sixth singlet excited states. The analysis of the dominant electronic configurations of those excited states reveals that all of them are essentially the result of single electron excitations from the iron 3d orbital to π orbitals on the phenyl fragments of the P_2_P^Ph^ ligand. Additionally, to check the effect of the dynamical correlation on the estimation of the absorption spectrum of complex **1**, we used NEVPT2 calculations to evaluate the energy of the quoted low-lying roots. As shown in Figure S1, the dynamical correlation has only a minor impact on the spectrum; the main bands showed a slight blue shift with respect to the CASSCF bands. The performance of the TD-DFT approach on the simulation of the absorption spectrum of **1** has also been tested, which seems to have some agreement with the experimental one (Figure S1). However, the large number of required roots and the great delocalization of the molecular orbitals make it difficult to assign the calculated wavelengths to the UV–vis bands as well as to determine the nature of the dominant electronic transitions. The agreement between CASSCF/NEVPT2 and experimental absorption bands evidences the reliability of the WFT-based approaches in the description of the relative energies and electronic structure of these complexes.

**Table 2:**
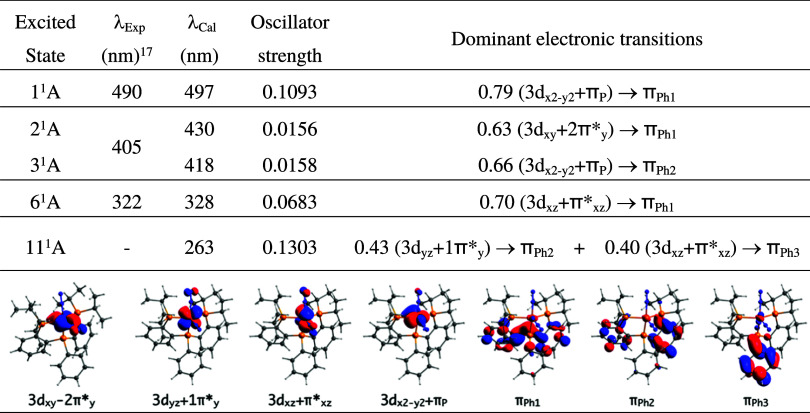
Absorption Wavelengths at the CASSCF(10,10) Level Associated with the Excitations with Larger Oscillator Strength of Complex **1**^a^

a The dominant electronic transition (last column) and the hole and particle involved in the electron transitions (last row) are also displayed.

Additionally, we studied the electronic structure of adducts **3** and **4**, which are four-coordinate complexes, where the central iron is in a distorted tetrahedral field (Scheme 2). A set of exploratory calculations indicated that the Fe 3d_*xy*_ and 3d_*xz*_ orbitals strongly interact with the π*_y_ and π*_z_ orbitals of the single N_2_ molecule. The remaining three 3d orbitals interact with four MOs of the P_2_P^Ph^ ligand (one σ-occupied and three π unoccupied orbitals). Therefore, we used CAS(11,11) and (12,11) for **3** and **4**, respectively, on account of the five 3d orbitals of Fe, the two N_2_ π* orbitals, and the four orbitals of the P_2_P^Ph^ ligand. The energies calculated at the CASSCF level for **3** and **4** indicate a ground doublet (*S* = 1/2) and singlet (*S* = 0) state, respectively; the corresponding low-lying excited states (quartet and triplet states, respectively) are separated by 51.0 and 81.6 kcal·mol^–1^.

In complex **3**, the analysis of the dominant contribution to the doublet ground state wave function (82% of |(σ_P_)^2^(3d_*yz*_ + π_*z*P_)^2^(3d_*xz*_ + π*_z_)^2^(3d_*x*^2^–*y*^2^_ + π_*x*P_)^2^(3d_*xy*_ + π*_y_)^2^(3d_*z*^2^_ + π_*y*P_)⟩) reveals that the gained electron by the reduction of **1** goes mostly to the orbitals of the P_2_P^Ph^ fragment, which in turn enhances the back-bonding toward the single N_2_ molecule (Figure S3). The fragment occupations of complex **3** resulting from the active NO decomposition give 3.9 electrons on the P_2_P^Ph^ ligand, 6.1 electrons on the Fe 3d orbitals, and 1.0 electrons on the N_2_ π* orbitals (Table S1). These occupations are consistent with a (P_2_P^Ph^)^2–^Fe^2+^–N_2_^1–^ species, where the oxidation state of each fragment just highlights the back-bonding from metal to the ligands, as in complex **1**. This corresponds to charge transfer, but it does not introduce a spin density on the fragments. Namely, the dinitrogen is highly activated, more than in complex **1**, as suggested by the significant reduction of the experimental N–N frequency (*v̅*_N–N_ = 1872 cm^–1^), compared to 2358.6 cm^–1^ for the isolated molecule.

A similar pattern is found in complex **4**. The main contribution (78%) to the ground singlet state wave function is |(σ_P_)^2^(3d_*z*2_ + π_*y*P_)^2^(3d_*yz*_ + π_*z*P_)^2^(3d_*x*^2^–*y*^2^_ + π_*x*P_)^2^(3d_*xy*_ + π*_y_)^2^(3d_*xz*_ + π*_z_)^2^⟩, where the two additional electrons with respect to **1** increase the population of the P_2_P^Ph^ fragment (Figure S4), reinforcing even more the back-bonding to N_2_. In fact, the occupations resulting from the active NOs (Table S2) amount to 1.2 electrons on the N_2_ molecule, which agrees with the lessening of the N–N frequency observed experimentally (*v̅*_N–N_ = 1677 cm^–1^). The oxidation states of the fragments in **4** can be roughly stated as (P_2_P^Ph^)^3–^Fe^2+^–N_2_^1–^.

### Dimerization Mechanism of (P_2_P^Ph^)Fe(N_2_)_2_

**Scheme 4:**
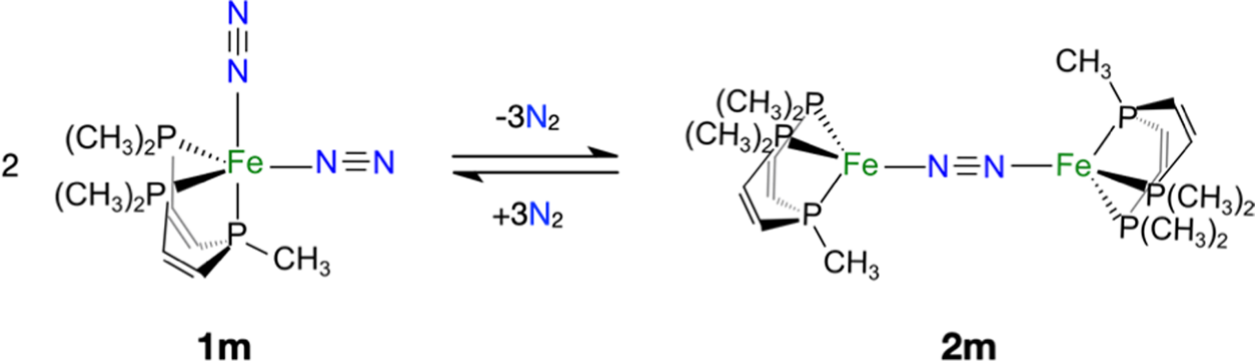
Chemical Equation (Including Structures) of the Equilibrium for the Dimerization from **1m** and **2m**

Several experimental data support the presence of a chemical equilibrium between the mononuclear complex **1** and the dinuclear mono-N_2_-bridged complex **2** (Scheme 2).^17^ Particularly, the formation of complex **2** from **1** was monitored by the absence of one N_2_ stretching frequency in the infrared (IR) spectrum, the release of N_2_ measured by a Toepler pump experiment, and the chemical shifts in the ^1^H and ^31^P NMR spectra.^17^ We proposed a dimerization mechanism from **1** to give **2** by means of DFT calculations. To make the calculations feasible in a moderate time frame, model complexes of **1** and **2** (**1m** and **2m**, Scheme 4 and Figure S4) are employed in all sets of calculations. Since the conversion between **1** and **2** is expected to involve notorious changes mainly in the geometrical parameters around the coordination sphere of Fe atoms, a small-scale model of the P_2_P^Ph^ ligand has been used by reducing only the external substituents (see the Computational Methods Section for details).

**Figure 2:**
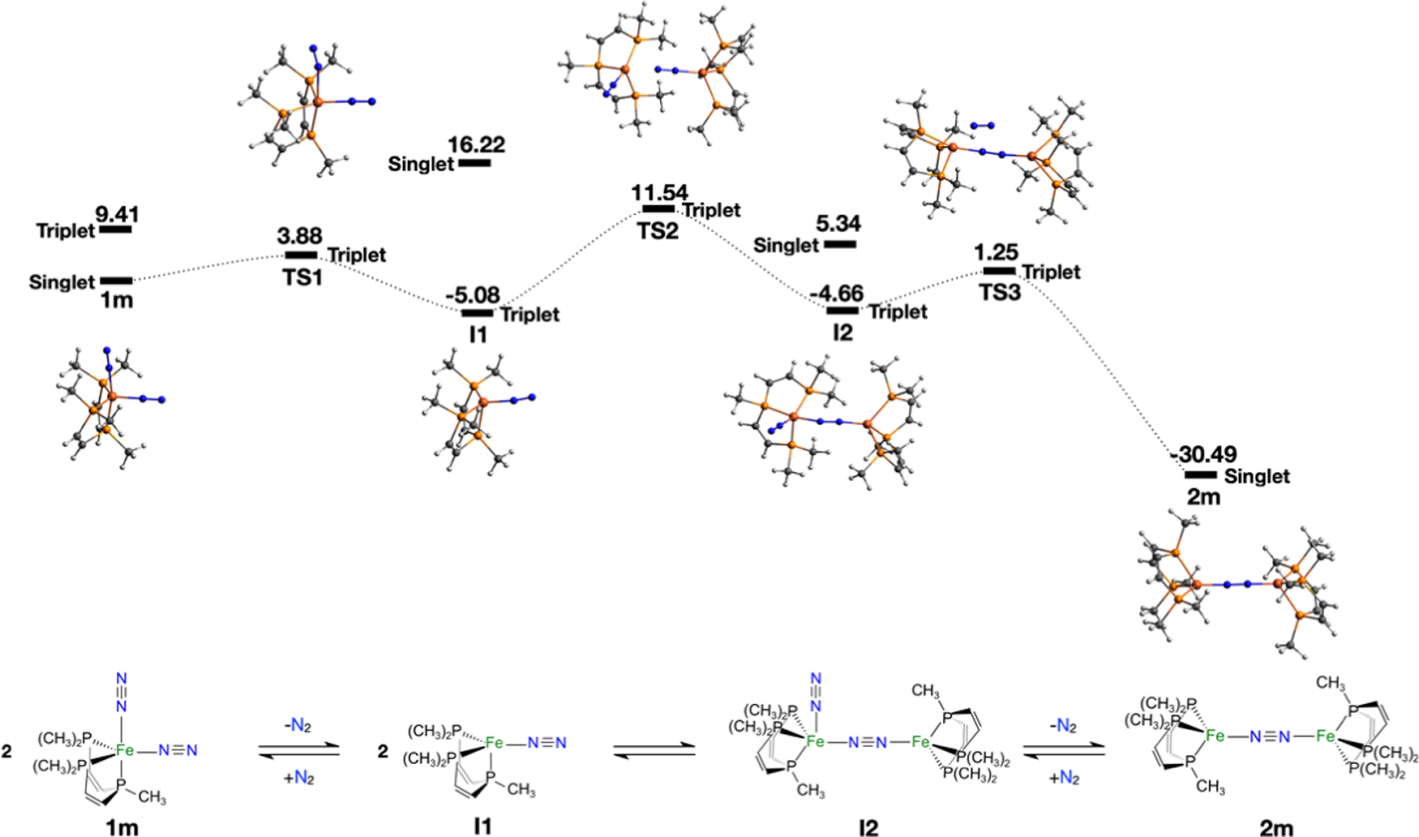
Gibbs free energy profile (kcal·mol^–1^) at 298 K for the dimerization of **1m** to give **2m** considering the stoichiometry. Ball and stick depictions of optimized geometries of intermediates and TS are also shown. Geometrical parameters around the Fe and N atoms in **1m**, **2m**, and the corresponding stationary points are outlined in Table S1.

The dimerization energy is estimated as Δ*E*_dimer_ = *E*(**2m**) + 3*E*(N_2_) – 2*E*(**1m**), according to the stoichiometry in Scheme 4. At the PBEh-3c level, both **1m** and **2m** have a singlet ground state and the dimerization energy is Δ*E*_dimer_ = −4.21 kcal·mol^–1^. The dimerization enthalpy (Δ*H*_dimer_ = −9.19 kcal·mol^–1^) and Gibbs free energy (Δ*G*_dimer_ = −30.49 kcal·mol^–1^) at 298 K have also been estimated. These values indicate that the formation of **2m** is exothermic and exergonic and therefore thermodynamically favored. Comparing Δ*G*_dimer_ with Δ*H*_dimer_, it follows that the entropic correction has a noteworthy impact on the process; therefore, the Gibbs free energies are used in the description of the dimerization energy profile. Optimized geometries of reactants, products, intermediates, and transition states (TS) are depicted in Figure 2 (Cartesian coordinates of the optimized geometries are reported in the SI file, and the main geometrical parameters are collected in Table S3). We propose a dimerization mechanism in three steps (Figure 2, bottom). In the first step, the axial N_2_ molecule is released from **1m** to achieve a triplet state intermediate of the Fe–N_2_ type (**I1**) at −5.08 kcal·mol^–1^ with respect to **1m** (the corresponding singlet state is 21.3 kcal·mol^–1^ above the triplet; Figure 2), which indicates an exergonic stage. In fact, four-coordinated species as **I1** have also been proposed as a transient intermediate on the H_2_ elimination from **pre-1** to produce **1**.^16,17^ The change in the multiplicity from **1m** to **I1** likely arises from the minimum energy crossing point (MECP) between the lowest triplet and singlet potential energy surfaces. Similar behavior is obtained with functionals of different nature and Fock exchange contribution, as shown in Table S5. The stability of the triplet state for **I1** is then not related to the functional but to the scission of one N_2_ molecule that weakens the Fe ligand field, favoring the high-spin state. Considering the triplet transition state found for this step (**TS1** with Δ*G*_TS1_ = 3.88 kcal·mol^–1^ with respect to **1m**), a low energy barrier is involved in the cleavage of the Fe–N bond (*d*_Fe–N_ = 2.32 Å in **TS1**). The second step is bimolecular; the interaction of two **I1** molecules gives rise to the formation of the N_2_–Fe···N_2_–Fe bonding at 2.50 Å as found in the triplet transition state **TS2** (Δ*G*_TS2_ = 11.54 kcal·mol^–1^ with respect to **1m**). Afterward, the triplet state intermediate **I2** is reached by −4.66 kcal·mol^–1^ with respect to reactants. The corresponding singlet state is 10 kcal·mol^–1^ above the triplet one (Figure 2). The formation of dinuclear intermediate **I2**, as usual for bimolecular reaction steps, is the rate-determining step of the reaction, which is evidenced by its higher energy barrier (Δ*G*^†^ = Δ*G*_TS2_). In fact, according to the Eyring–Polányi equation^22^, we estimate a rate constant of *k* = 2.16 × 10^4^ s^–1^ M^–1^ at *T* = 298.15 K, in line with the rapid dimerization suggested by the NMR and IR spectra,^17^ and the rate constant estimations in similar systems.^23^ Finally, the cleavage of a second N_2_ molecule takes place via a triplet **TS3** with a low barrier of 5.91 kcal·mol^–1^ regarding **I2**, which ultimately leads to the Fe–N_2_–Fe complex (**2m)** placed at −30.49 kcal·mol^–1^ with respect to the reactants.

Overall, the calculated energy for each step of the reaction as well as its corresponding energy barrier suggests the reversibility of the dimerization process even at room temperature. In fact, this suggestion agrees with the experimentally observed equilibrium between **1** and **2** based on the changes observed in the signals of the ^1^H NMR measurements before and after degassing the solution.^17^

### Magnetic Coupling Constant of the Dinuclear Species

As stated before, the formation of the diiron complex **2** has been followed by ^1^H and ^31^P NMR spectroscopies.^17^ A remarkable deviation from the Curie behavior in the ^1^H and ^31^P chemical shifts as a function of 1/*T* was observed.^17^ The magnetic interaction between the two *S* = 1 Fe centers can be modeled through a Heisenberg–Dirac–van Vleck Hamiltonian, Ĥ_HDVV_ = −2*J*Ŝ_1_·Ŝ_2_, where *J* corresponds to the magnetic coupling constant (*J* > 0 for ferromagnetic coupling, *J* < 0 for antiferromagnetic coupling). The singlet ground state of **2** results from the antiferromagnetic coupling of the Fe sites, and the deviation from Curie behavior can be related to the population of the excited triplet and quintet states, separated by 2*J* and 6*J*, respectively, from the singlet ground state. The *J* value can be obtained from fitting of the corresponding Boltzmann function to the experimental chemical shifts. The fitting yielded *J* = −940 cm^–1^; hence, a significant antiferromagnetic coupling governs the interaction between the two Fe centers in complex **2**. Comparable antiferromagnetic behavior has also been observed in other N_2_-bridged diiron species.^24,25^

**Figure 3:**
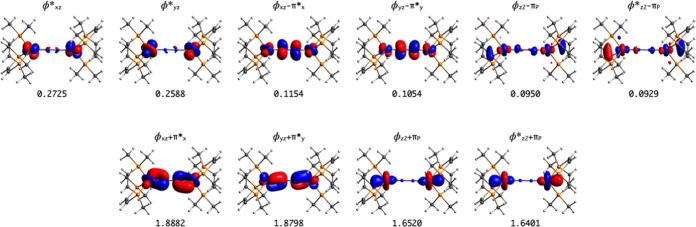
Active natural orbitals of N_2_-bridged diiron complex **2m**. Occupations of the singlet ground state are included.

We have evaluated the exchange coupling constants from the energy difference between the ground singlet state and the quintet state at the CASSCF and NEPVT2 levels. To reduce the computational cost, the model **2m** system is employed with an active space CAS(8,10). In the complex, each iron atom presents a distorted tetrahedral field, where only the 3d_*xz*_ and 3d_*yz*_ orbitals of each Fe exhibit a strong interaction with the N_2_ π*_x_ and π*_y_ orbitals (in the **2m** model, the Fe–N–N–Fe axis is parallel to the *z* axis). The Fe 3d_*z*^2^_ orbital slightly interacts with the unoccupied π orbital of the P_2_P^Ph^ ligand (π_P_), while the Fe 3d_*xy*_ and 3d_*x*^2^–*y*^2^_ orbitals do not interact with the N_2_ orbitals and are doubly occupied in the explored magnetic states. Figure 3 shows the active natural orbitals of the **2m** complex with the occupation numbers of the singlet state. In this figure, ϕ = 3d + 3d and ϕ* = 3d – 3d are the bonding and antibonding combination of the 3d orbitals of the two Fe atoms. The dominant contributions to the CASSCF(8,10) wave functions of the ground singlet and quintet states are
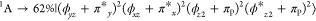

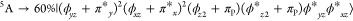
The linearity of the N_2_ bridge in complex **2m** and the high degree of Fe–N_2_ covalency favor the stabilization of the singlet state. Based on the CASSCF energies of the studied singlet and quintet states, the estimated coupling constant is *J* = −1530 cm^–1^, which agrees with the sign and amplitude of the fitted *J* value. Since the dynamical correlation has been proved to be critical to accurately estimate the energetic separation of the magnetic states,^26,27^ we have also performed NEVPT2 calculations on **2m** using the CASSCF wave functions as the zeroth order reference. At this correlated level, the calculated *J* value is −640 cm^–1^, which is also in line with the predicted AF nature. Despite the ligand simplification in **2m**, we found that the calculated *J* value agrees well with the amplitude and sign of *J* estimated for **2** from NMR experiments.^17^

## Conclusions

In this work, we have carried out a study of the N_2_ activation in a set of phenylphosphine–Fe complexes, the neutral complex (P_2_P^Ph^)Fe(N_2_)_2_ (**1**), the anionic ones [(P_2_P^Ph^)Fe(N_2_)]^−^ (**3**) and [(P_2_P^Ph^)Fe(N_2_)]^2–^ (**4**), and the dinuclear mono-N_2_-bridged complex [(P_2_P^Ph^)Fe]_2_(μ–N_2_) (**2**) by means of multiconfigurational WFT-based calculations. The electronic structures of these species have been analyzed in terms of the occupation of the Fe, N_2_, and P_2_P^Ph^ fragments in the active natural orbitals. For those ionic species, our calculations revealed the enhancement of the N_2_ activation, reaching a reduction to N_2_^1–^, due to the back-bonding from the Fe atoms, in agreement with the attenuation of the N–N vibrational frequencies observed experimentally. Therefore, the oxidation state of mononuclear ions can be better understood as (P_2_P^Ph^)^2–^Fe^2+^–N_2_^1–^ and (P_2_P^Ph^)^3–^Fe^2+^–N_2_^1–^ species, whereas the dinuclear complex as (P_2_P^Ph^)^1–^Fe^2+^–N_2_^2–^–Fe^2+^(P_2_P^Ph^)^1–^ one, in accordance with our multiconfigurational calculations.

A mechanism for the dimerization between **1** and **2** (using models **1m** and **2m**) has also been proposed within the framework of the DFT approach. The energy profile of the dimerization indicates a mechanism in three steps, where the formation of an Fe–N_2_···Fe adduct is the rate-determining step of the reaction (Δ*G*^†^ = 11.5 kcal·mol^–1^). The formation of dimer **2m** is a thermodynamically favored process (Δ*G*_dimer_ = −30.5 kcal·mol^–1^). Moreover, we estimated the magnetic coupling constant of complex **2m** using the NEVPT2 approach. **2m** behaves as a strong molecular antiferromagnet (*J* = −640 cm^–1^), which is explained in terms of two *S* = 1 Fe d^6^ centers, coupled through the superexchange pathway provided by the bridging N_2_ ligand. Work is in progress to analyze the photochemical process involving some of these phenylphosphine–Fe complexes.

## Computational Methods

### Benchmark Models and Computational Details

To evaluate the electronic structure of complexes **1**–**4**, we have carried out single-point calculations by means of the CASSCF^28^ and NEVPT2^29^ methods. Specific details about the application of these wave function theory (WFT)-based approaches are described below. The geometries used for these calculations (except for **2**) were reported by Peters et al., as included in the CCDC with identification codes p17278, p17634Pnma, and p17581, respectively.^17^ To validate the use of crystallographic structures, we performed geometry optimization calculations of complex **1** with both density functional theory (DFT) and wave function-based approaches. These calculations do not reveal significant changes in the bond parameters of the Fe center (Table S4). Apart from that, to determine the dimerization mechanism between complexes **1** and **2**, we have used model systems. In these models, the external substituents of the P_2_P^Ph^ ligand have been simplified (Figure S4): the bridging orthophenylene groups have been reduced to 1,2-ethene, and the isopropyl and phenyl groups have been replaced by methyl groups. Geometry optimization calculations have been carried out on **1m** and **2m** on the DFT framework using the M062X, TPSSh, and PBEh-3c functionals; the latter being a counterpoise corrected version of the PBE0 functional (vide infra). Our results indicate that the PBEh-3c functional shows a better performance in the description of relative energies, e.g., the dimerization energy at the TPSSh level indicates an unfavorable process, while the dimerization energy is overestimated at the M062X level, against the reversibility of the process and the observed coexistence of mononuclear and dinuclear species. However, the PBEb-3c functional shows a balance between these edging behaviors, leading to a dimerization energy consistent with a favorable and reversible process (Table S5). PBEh-3c has also exhibited good performance in the search for transition states of our model complexes (Table S5), as it was also reported in comparable compounds.^30^

In the entire study, the all-electron Ahlrichs basis sets were used as follows: the def2-TZVP contraction for Fe atoms; for atoms directly coordinated to the metal, P, and N, the def2-TZVP(-f) contraction has been chosen; for C and H, the basis set is reduced to def2-SVP.^31^ The resolution of identity approach has also been considered, by using the def2/J auxiliary basis set, in the computation of Coulomb integrals.^32^ In DFT calculations, it is also included the long-range interactions by the Grimme correction to the energy (D3BJ)^33^ and the Douglas–Kroll–Hess (DKH) relativistic correction to the basis and Hamiltonian,^34^ as implemented in the ORCA package. The threshold for the energy convergence in the SCF procedure is 1 × 10^–8^ a.u. The analytical frequencies of stationary points along the dimerization between **1m** and **2m** have also been calculated. No negative frequencies were obtained on the optimized geometries, except for the saddle points characterized by a large imaginary frequency. All calculations were performed with the ORCA 4.2.1 package.^35,36^ In CASSCF/NEVPT2 calculations, a Gaussian fitting with a line width of 50 nm was used for plotting the spectrum curves. The Avogadro visualization tools^37^ have been used for plotting geometries and orbitals, as well as to animate the vibrational normal modes.

### Strategy for WFT-Based Calculations

We performed CASSCF calculations to analyze the electronic structure of complexes **1**, **2m**, **3**, and **4**. The active space has been rationally selected to reduce as much as possible the high computational demands without losing the validity of the findings. Some computational experiments with different CAS sizes led us to set up the minimal active space as follows

- A CAS(10,10) for **1**, including the five 3d atomic orbitals of Fe, three π* MOs of two N_2_ molecules, and two orbitals of the P_2_P^Ph^ ligand centered on the P atoms.
- A CAS(8,8) for **2m**, where the three higher energy 3d orbitals of each iron atom have been included as well as the two π* MOs of the N_2_ moiety.
- A CAS(11,11) and CAS(12,11) for **3** and **4**, respectively, including the five Fe 3d orbitals, the two N_2_ π* MOs, and four orbitals on the P_2_P^Ph^ ligand.

To obtain the oxidation states of the P_2_P^Ph^, Fe, and N_2_ moieties, we have derived their partial populations by decomposing the occupation of each active orbital according to the normalized contribution of the atomic orbitals of the different fragments to each MO. Thus, the occupations of the three fragments per MO are obtained. If a fragment has an occupation lower than 0.5, the charge is taken as 0. For a fragment occupation between 0.5 and 1.0, we attribute 1 negative charge. Two negative charges are assigned for occupations larger than 1.0. In every case, we give priority to the Fe atom. We have previously used a similar strategy to evaluate the activation of O_2_,^38−40^ the magnetic coupling of dinuclear systems,^41,42^ as well as other kinds of problems.^43,44^

### Particulars of DFT-Based Calculations

To figure out the mechanism of the dimerization of **1**, we have performed geometry optimization calculations on the **1m** and **2m** models and on several potential intermediates at the PBEh-3c level with different multiplicities (2*S* + 1 = 1, 3, and 5). This functional is a variant of the PBE functional with a relatively large amount of nonlocal Fock exchange (42%) and a valence-double-ζ Gaussian AO basis set (def2-mSVP).^45^ PBEh-3c is particularly well suited in the optimization of geometries and in the estimation of interaction energies of noncovalent complexes.^30^ The basis set superposition errors and London dispersion effects are also accounted for. Additionally, to find the transition state (TS) structures, some relaxed scans on different Fe–N bond lengths have also been realized for *S* = 0, 1, and 3 states. At the state with the lowest energy, we choose the higher energy structure to optimize the TS geometry. To display the energy profile of the dimerization, we have taken into consideration the stoichiometry of the reaction and the relative energies of intermediates, TSs and products were computed with respect to the reactants as in Scheme 4.

## Supporting Information Available

Simulated UV–vis spectra with different approaches; active orbitals of complexes, **1**, **3**, and **4**, and structures models for **1m** and **2m**; analysis of the wave function of complexes **3** and **4**; geometrical parameters for the intermediates and additional calculations with different functionals for the dimerization pathway; and Cartesian coordinates for the optimized geometries of all considered models (PDF)
