# Supplementary material for: Dinitrogen Activation Mediated by the (P2PPh)Fe Complex: Electronic Structure, Dimerization Mechanism, and Magnetic Coupling
Source: Inorg Chem. 2024 Jan 9;63(3):1633–41. doi: 10.1021/acs.inorgchem.3c03813 (PMC10954229; doi:10.1021/acs.inorgchem.3c03813)
Supplement: Supplementary file 1 — ic3c03813_si_001.pdf [file ic3c03813_si_001.pdf]

Supporting Information

Dinitrogen Activation Mediated by the (P_2P^{Ph})Fe Complex: Electronic Structure, Dimerization Mechanism and Magnetic Coupling

Jhon Zapata-Rivera,[†*] Carmen J. Calzado[‡*]

[†] Facultad de Ciencias Naturales y Exactas, Departamento de Química, Universidad del Valle.

Calle 13 N° 100 – 00. 25360, Cali, Colombia. jhon.zapata.rivera@correounivalle.edu.co

[‡] Departamento de Química Física. Universidad de Sevilla. c/ Profesor García González, s/n.

41012, Sevilla. Spain. calzado@us.es

Contents

Figure S1. Simulated UV-vis absorption spectrum of complex **1** based on (a) CASSCF(10,10), (b) CASSCF(10,10)/NEVPT2, and (c) TD-DFT TPSSh calculations.

Figure S2. Active molecular orbitals and occupations of complex **3**.

Table S2. Normalized contribution (NC) of the P_2P^{Ph}, Fe, and N_2 fragments to the active MOs of the singlet ground state of complex **3**.

Figure S3. Active molecular orbitals and occupations of complex **4.**

Table S2. Normalized contribution (NC) of the P_2P^{Ph}, Fe, and N_2 fragments to the active MOs of the singlet ground state of complex **4**.

Figure S4. Depiction of isomerization between **1** and **2**. Structures of model complexes **1m** and **2m** are included.

Table S3. Geometrical parameters around the Fe and N atoms in **1m**, **2m**, and stationary points in their corresponding ground states.

Table S4. Geometrical parameters surrounding the Fe center of complex 1 at the PBEh-3c, TPSSh and CASSCF(4,3) level.

Table S5. Relative energies(kcal·mol^{-1}) of the triplet state with respect to the monomer 1m for some stationary points in the dimerization pathway between **1m** and **2m** with different functionals.

Table S6. Cartesian coordinates of the optimized PBEh-3c geometries and imaginary frequencies characterizing the transition states.

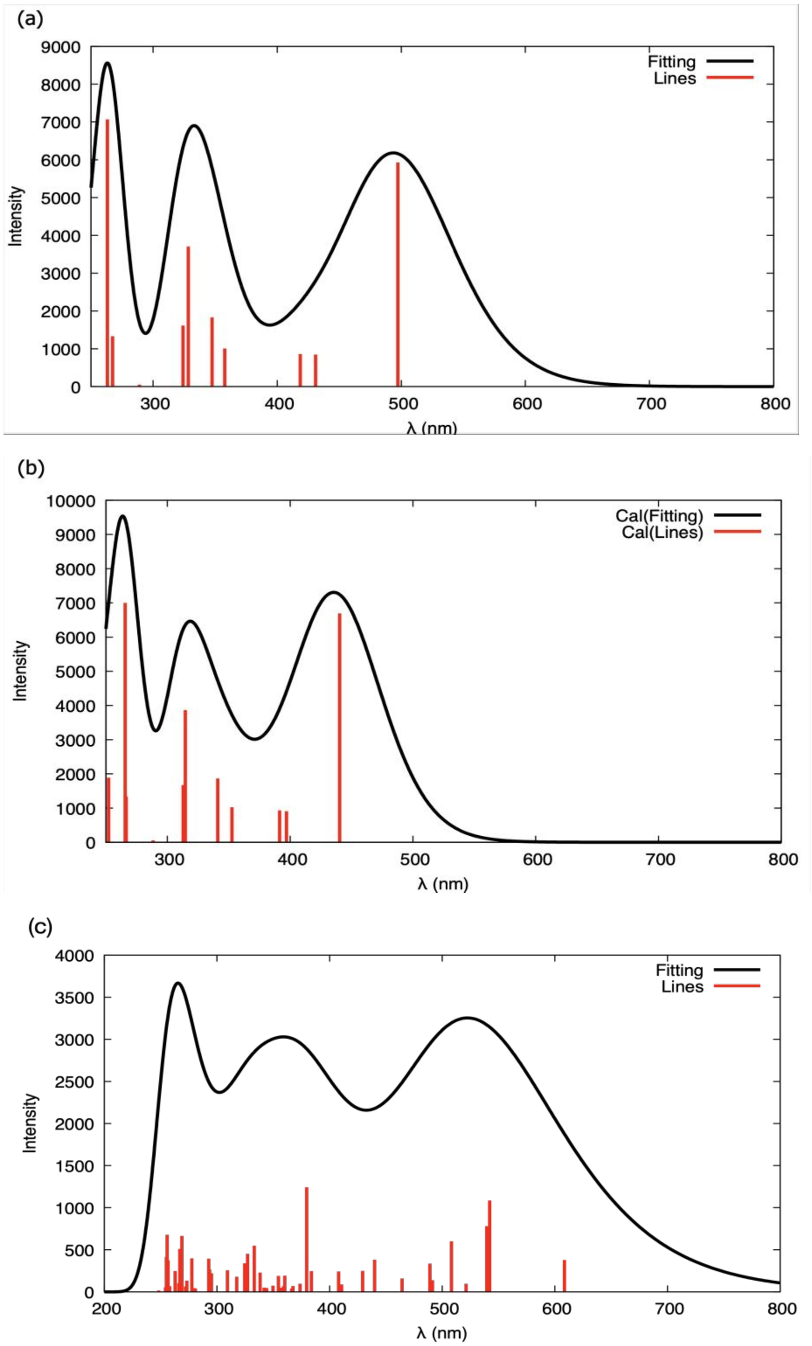

Figure S1. Simulated UV-vis absorption spectrum of complex **1** based on (a) CASSCF(10,10), (b) CASSCF(10,10)/NEVPT2, and (c) TD-DFT TPSSh calculations.

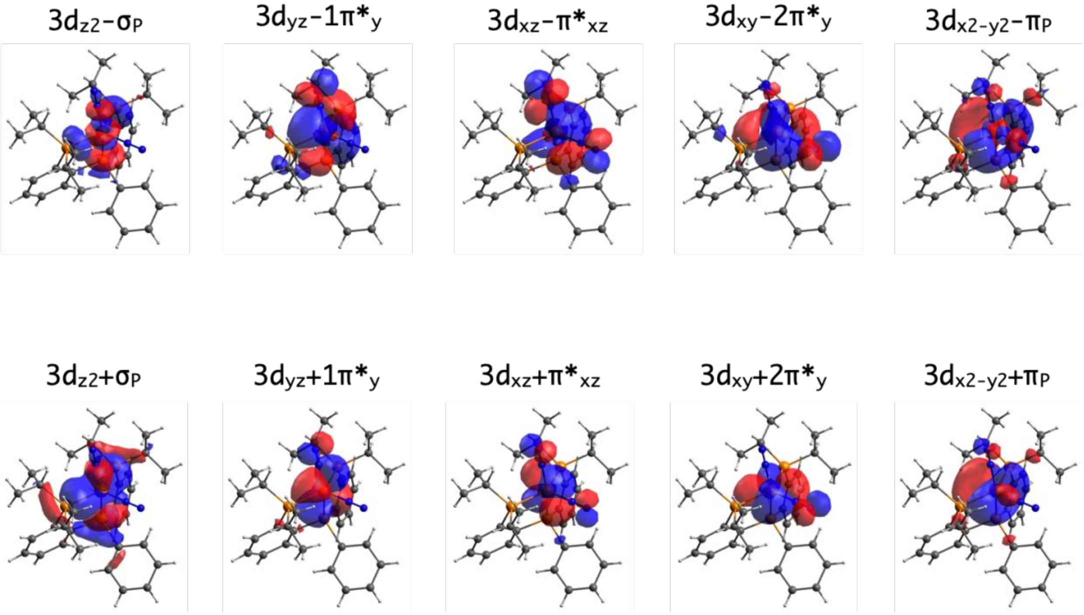

Figure S2. Active molecular orbitals of complex **1** with isosurface value of 0.025.

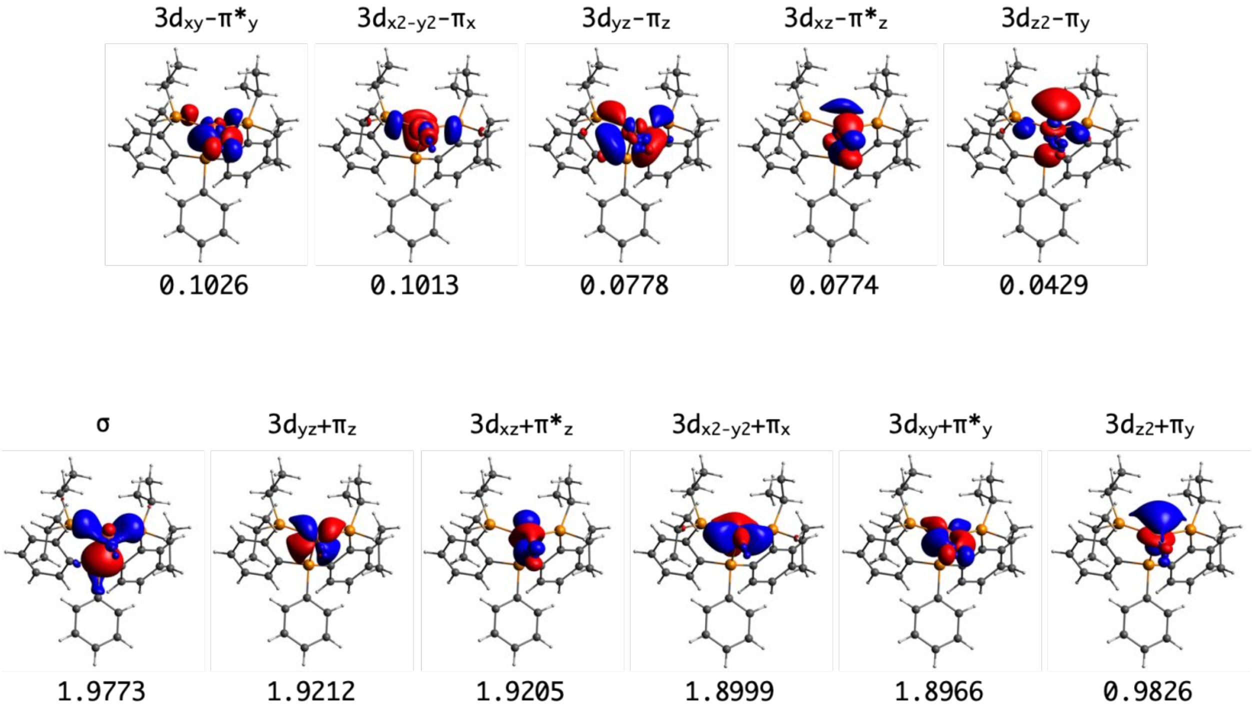

Figure S3. Active molecular orbitals and occupations of complex **3**.

Table S1. Normalized contribution (NC) of the P_2P^{Ph}, Fe, and N_2 fragments to the active MOs of the singlet ground state of complex **3**. The corresponding fractional occupation (occ) per fragment is also included.

	Active MO Occupations	1.98	1.92	1.92	1.90	1.90	0.98	0.10	0.10	0.08	0.08	0.04	Occ per fragment
NC	Fe	0.09	0.79	0.79	0.45	0.65	0.60	0.51	0.48	0.50	0.53	0.53	
	N_2	0.02	0.01	0.06	0.11	0.11	0.24	0.34	0.15	0.07	0.11	0.01	
	P_2P^{Ph}	0.89	0.10	0.10	0.44	0.20	0.36	0.26	0.44	0.47	0.23	0.43	
Occ	Fe	0.18	1.52	1.52	0.86	1.23	0.59	0.05	0.05	0.04	0.04	0.02	**6.10**
	N_2	0.05	0.20	0.20	0.20	0.29	0.04	0.02	0.01	0.00	0.02	0.00	**1.04**
	P_2P^{Ph}	1.75	0.19	0.19	0.84	0.38	0.35	0.03	0.04	0.04	0.02	0.02	**3.86**

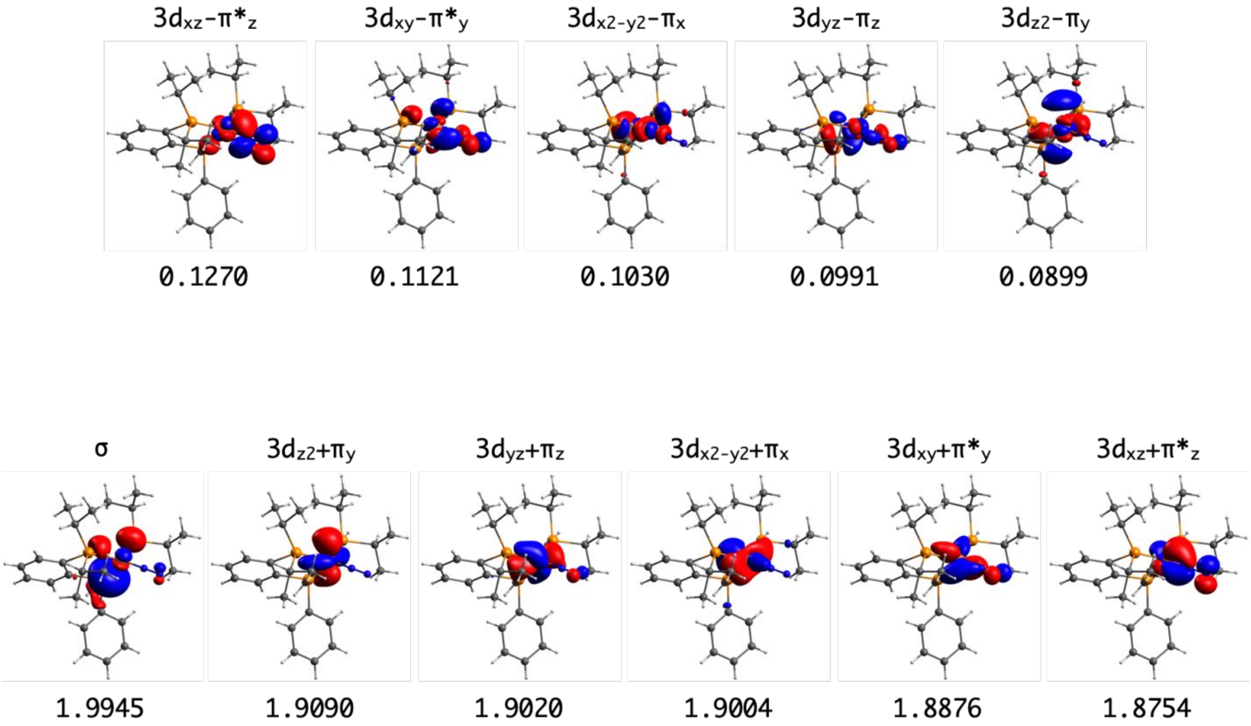

Figure S4. Active molecular orbitals and occupations of complex **4**.

Table S2. Normalized contribution (NC) of the P_2P^{Ph}, Fe, and N_2 fragments to the active MOs of the singlet ground state of complex **4**. The corresponding fractional occupation (occ) per fragment is also included.

Active MO Occupations	1.99	1.91	1.90	1.90	1.89	1.88	0.13	0.11	0.10	0.10	0.09	Occ per fragment

	Fe	0.11	0.63	0.59	0.41	0.55	0.55	0.45	0.42	0.49	0.44	0.43	
NC	N_2	0.02	0.01	0.06	0.13	0.11	0.24	0.34	0.15	0.07	0.11	0.01	
	P_2P^{Ph}	0.87	0.37	0.35	0.45	0.34	0.20	0.21	0.43	0.44	0.45	0.56	
	Fe	0.21	1.20	1.12	0.78	1.04	1.03	0.06	0.05	0.05	0.04	0.04	**5.63**
Occ	N_2	0.04	0.01	0.11	0.25	0.21	0.46	0.04	0.02	0.01	0.01	0.00	**1.17**
	P_2P^{Ph}	1.74	0.70	0.67	0.86	0.64	0.38	0.03	0.05	0.04	0.04	0.05	**5.20**

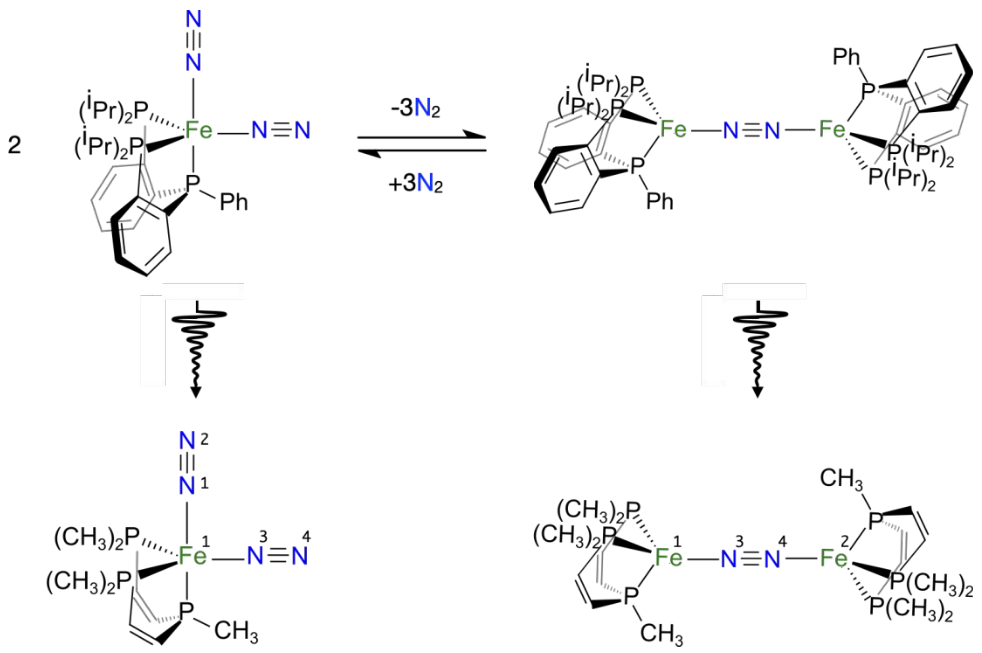

Figure S4. Depiction of isomerization between **1** and **2**. Structures of model complexes **1m** and **2m** are included.

Table S3. Geometrical parameters around the Fe and N atoms in **1m**, **2m**, and stationary points in their corresponding ground states. Labels as in Figure S4.

	1m	TS1	I1	TS2	I2	TS3	2m
d(N1-N2)	1.106	1.096	-	1.103	1.103	1.098	-
d(Fe1-N1)	1.801	2.318	-	1.824	1.838	2.589	-
a(Fe1-N1-N2)	178.7	159.1	-	178.5	179.3	136.2	-
a(N1-Fe1-N3)	92.5	87.4	-	86.7	89.9	72.9	-
d(N3-N4)	1.108	1.109	1.115	1.163	1.178	1.175	1.184
d(Fe1-N3)	1.796	1.876	1.832	2.500	1.850	1.774	1.767
d(Fe2-N4)	-	-	-	1.776	1.768	1.783	1.767
a(Fe1-N3-N4)	179.9	177.2	172.6	126.7	151.7	170.7	178.000
a(Fe2-N4-N3)	-	-	-	172.7	175.9	171.8	178.500
h(Fe1-N3-N4-Fe2)	-	-	-	185.7	258.1	321.3	206.800

Table S4. Geometrical parameters surrounding the Fe center of complex 1 at the PBEh-3c, TPSSh and CASSCF(4,3) level. The reference values from the solid-state crystal are also reported. Bond distances in Å, bond angles and dihedrals angles in degrees.

	Crystal X-ray Data	PBEh-3c Singlet	TPSSh Singlet	CASSCF(4,3) Singlet
b(N1-N2)	1.110	1.130	1.128	1.074
b(N3-N4)	1.102	1.133	1.130	1.075
b(Fe-N1)	1.881	1.790	1.800	2.179
b(Fe-N3)	1.883	1.788	1.801	2.142
b(Fe-P1)	2.190	2.155	2.148	2.342
b(Fe-P2)	2.242	2.173	2.166	2.419
b(Fe-P3)	2.234	2.170	2.163	2.380
a(N1-Fe-N3)	88.600	91.800	91.100	84.800

a(N1-Fe-P1)	177.200	175.500	175.800	177.100
a(N1-Fe-P2)	93.300	92.600	92.700	94.900
a(N1-Fe-P3)	93.400	93.100	93.200	95.000
a(N3-Fe-P1)	94.100	92.700	93.100	98.100
a(N3-Fe-P2)	106.400	114.600	114.300	115.300
a(N3-Fe-P3)	104.100	116.000	116.000	110.000
a(P1-Fe-P2)	85.200	85.200	85.300	83.800
a(P1-Fe-P3)	86.700	85.300	85.300	84.000
a(P2-Fe-P3)	149.000	128.800	129.200	134.200

Table S5. Relative energies(kcal·mol^{-1}) of the triplet state with respect to the monomer 1m for some stationary points in the dimerization pathway between **1m** and **2m** with different functionals. Each system has been independently optimized for each functional and state. Values in parenthesis belong to the singlet state. We were unable to find TS1 with the M062X functional.

Functional	ΔE_{TS1}	ΔE_{Int1}	ΔE_{dimer}
PBEh-3c	9.45	10.75 (32.06)	-4.21
TPSSh	19.66	24.46 (36.34)	32.16
M062X	-	1.87 (29.07)	-62.10

Table S6. Cartesian coordinates of the optimized PBEh-3c geometries and imaginary frequencies characterizing the transition states.

1m, singlet

Fe	12.20565130194344	5.09905642021815	11.35493532063247
P	10.38329047150312	5.04396902517464	10.20096996356186
P	12.53838524263778	3.13083812198274	10.59401273339073
P	12.72160278837905	6.77158441578440	10.13119016768693
N	11.33319428001870	5.34167832746174	12.90551303091895
C	10.37177944273614	6.31183311670744	8.90233881381959
C	8.72080679176537	5.22016471278207	10.92698118891520
N	10.79328661974068	5.49160480874030	13.86190120136533

C	11.44685018710465	7.09768908610015	8.87521616844490
C	10.23061343148157	3.50080291451483	9.25842361441001
N	13.81410241870355	5.12206186147915	12.16587320156885
C	11.22241489286147	2.63086292095535	9.44161150603791
C	12.54684538558179	1.69991602950057	11.73991617898276
C	14.23601546605030	6.76987433765081	9.09907058339224
N	14.81197445436984	5.13558710507998	12.64169435574120
C	12.88759125153876	8.43256841759979	10.88895777626090
C	14.03225276025223	2.72663842171542	9.61245843527865
H	7.92838422249531	5.16702555880138	10.17135763825990
H	8.65108786771757	6.18278919892648	11.44510416037735
H	8.56001442982076	4.42645413972533	11.66463517408695
H	13.09035779466565	9.22684158356344	10.15905000671703
H	13.70914473821804	8.40921441771271	11.61501665700378
H	11.97048190182691	8.67093932961720	11.43762795520300
H	12.65502562548744	0.73409122331830	11.22972631080065
H	11.61916197616521	1.69922967718666	12.32157880832394
H	13.37812760575819	1.81535700391286	12.44584124982588
H	14.08104329371208	3.38132718370260	8.73618901899345
H	14.05960847514752	1.68093294259780	9.28093089674196
H	14.92457526026448	2.91843150019258	10.22029698584753
H	14.36622680920741	7.69532843187802	8.52394661850604
H	14.20610584922111	5.92075874212835	8.40833263207745
H	15.11176969204580	6.64080083755176	9.74634478219881
H	9.38611378768043	3.30158900196271	8.59305072459330
H	11.24875748490432	1.66337910116721	8.93125480946438
H	9.54230347891991	6.42157010245808	8.19840802697608
H	11.56162252007284	7.90011998014878	8.14031330359357

TS1, triplet, Freq=-128.76

Fe	12.11399963469646	5.16880260171623	11.57610725442311
P	10.22431345421370	5.13392384633583	10.30312636307334
P	12.51546600346195	3.16624747843750	10.66620047415296
P	12.78346492820503	6.82936651728870	10.15397432954683
N	11.43171262649817	4.73128731172453	13.26826642112551
C	10.43122443137969	6.27306187195431	8.89071227273934
C	8.44671967102378	5.40496814336644	10.66938875764543
N	11.01991092783387	4.52562111840235	14.27769813564164
C	11.54946453153675	6.99654152890959	8.82928514515110
C	10.13179424399711	3.54611813148653	9.40938372756813
N	14.22229195123460	5.14915027525890	12.53897074720219
C	11.13469820180770	2.68505402880189	9.57933148556423
C	12.63571519656064	1.68066139579659	11.73371802263741
C	14.33520210004191	6.78094241878632	9.18604178486260
N	15.29328494186175	4.94726794494556	12.65383575127635
C	12.84520935536154	8.55359740786859	10.77151183716413
C	13.92505738590505	2.83657349595997	9.53721835341515
H	7.79500962073689	5.29593125016463	9.79276547522119

H	8.31339883705086	6.41115241086382	11.08244394229470
H	8.12792188927276	4.68810131211359	11.43461320639135
H	12.98907702986601	9.28856893933957	9.96948547043591
H	13.66802929523582	8.65376152158838	11.48913935769325
H	11.91264958565155	8.78431223182749	11.29742322788123
H	12.67636856901988	0.74105891357545	11.16793454126055
H	11.77459143902259	1.65155092376319	12.41006791700381
H	13.53867010307265	1.75358814505458	12.35179139511794
H	13.88281822249745	3.53075764749482	8.69082090040346
H	13.93497794650004	1.81065916542347	9.14769965597944
H	14.86794001913979	3.01294920535409	10.06878548686081
H	14.42740848111802	7.62279007163822	8.48796044720211
H	14.38770458835077	5.84550918302672	8.61987626708378
H	15.19113665560372	6.80248964162348	9.87059606457655
H	9.28399338898016	3.31011402354157	8.75834549329051
H	11.14279795425280	1.71680009480857	9.06780445440404
H	9.66349069640373	6.36179790328116	8.11535229780858
H	11.72905609259901	7.69183189847489	8.00239353589629

I1, triplet

Fe	12.29682223242643	5.08619560020538	11.28240465901001
P	10.41121081821773	5.04111616206871	10.15397576971371
P	12.52566595311025	2.94603944674093	10.67728755313382
P	12.72668508552918	6.98343216057790	10.18369944440166
N	11.86744214377166	5.33204880339345	13.04616028554800
C	10.42914201623082	6.33012529183473	8.86415803275972
C	8.66930897940181	5.18833231954614	10.71514684133783
N	11.74687906245240	5.47729752012286	14.14470349386436
C	11.43822217595346	7.20091012195641	8.91146297514357
C	10.29721779924925	3.49150991291660	9.19965477610455
C	11.21714407131426	2.56325718122433	9.46588218411712
C	12.31510235495200	1.57955701664466	11.87193882095480
C	14.23235615002302	7.16577285977470	9.15605161855069
C	12.69151629385681	8.61301483261698	11.00965217021457
C	14.00026232523411	2.36234276287141	9.76052102591472
H	7.93764344682492	5.12652197526893	9.89942550615363
H	8.54295310751589	6.14707943861393	11.23039839103333
H	8.46075440099129	4.39091647091617	11.43712746989490
H	12.74947753089311	9.45095515749404	10.30321141028340
H	13.53409601161536	8.67925649061761	11.70739499239397
H	11.76961293520825	8.69898882322343	11.59382530271865
H	12.28102838494969	0.59375222679379	11.39071605896414
H	11.38937141970818	1.73644964648306	12.43487971175425
H	13.14578165530341	1.59633605718100	12.58663307303605
H	14.19496193828829	3.03112012232042	8.91510494774414
H	13.88798177034382	1.33865159789909	9.38146840149832
H	14.87397816859601	2.39569302176236	10.42152860825628
H	14.22075026148380	8.07537383070311	8.54220922939341

H	14.33323729127297	6.29719342881005	8.49629448040995
H	15.11358548654075	7.19554800323675	9.80720688425194
H	9.49581731513374	3.30377250471416	8.47837798530665
H	11.20133660904228	1.58708557579883	8.96948179655439
H	9.63648067282182	6.42148201104895	8.11513703335269
H	11.50707413174234	8.03148162461880	8.20079906622997

TS2, triplet, Freq=-107.34

Fe	13.60301957398146	16.96940306060102	4.42742365936746
Fe	10.22911295315883	16.18812478519369	7.61390035106866
P	13.46168960854664	18.74677335854742	2.99709585020775
P	15.71901802191632	17.70590011171871	4.74830168457478
P	14.63906884817528	15.18140442526499	3.45131158298205
P	8.32044806113100	15.58571830449122	8.43169897629591
P	9.52389816888248	18.21281712303578	7.87214199678378
P	11.09443778542442	15.33306498233929	9.40737560644413
N	10.00545883762425	16.69383923129789	3.35597118682871
N	10.11265707314983	15.61502838024508	3.45520710675359
N	12.37221617836201	16.65142131646837	5.66587106551185
N	11.51884187565468	16.40287093550937	6.44402514221220
C	16.05278183354015	19.16685654204975	3.70948867830263
C	15.05524395561078	19.62404919260759	2.95304844846879
C	12.31552352695777	20.11806613775897	3.38120480506249
C	13.12149826106121	18.53313646193238	1.21205690632454
C	16.92371780966871	16.50454791497441	4.09354150207907
C	16.44271983809664	15.38951795509656	3.54273175989937
C	16.57013418866567	18.20834028388908	6.29170793142332
C	14.41745965588500	13.54671255672129	4.23453008389599
C	14.42850065718707	14.73551297845331	1.68896501366987
C	7.30864046672190	17.04380418487393	8.85922720160362
C	7.85144529452575	18.23330989818480	8.59469577574037
C	8.53106335514361	14.79276075607456	10.06270891320585
C	9.78722408419730	14.67042454073558	10.49382705612370
C	7.06199427098931	14.49532584088964	7.67261603748731
C	9.30678914008238	19.21793064534314	6.36148298570755
C	10.38379151860727	19.43054381033718	8.93451543193470
C	12.20730838812646	13.88795458859624	9.25163483698442
C	12.05727806357191	16.33193795078711	10.60421975555421
H	12.52608371487743	13.47638597150400	10.21774570542496
H	11.70440931661085	13.10458078453470	8.67494572175265
H	13.09391330413605	14.19528217092085	8.68521638567507
H	10.01946371612336	14.20875770393529	11.45904836577318
H	7.68010222433613	14.43638005251356	10.65095092044803
H	11.44735804379814	17.17008209774311	10.95603032068758
H	12.39872763930282	15.75318177457671	11.47203921435103
H	12.93237434086786	16.74806388359110	10.09177305086302
H	8.63514566529842	18.69392415370593	5.67408996833320
H	8.91435195521193	20.22388939033427	6.55608098914288

H	10.28104436989813	19.30070639168992	5.86584972319521
H	9.88938337765363	20.41010580875838	8.95249310014507
H	10.44795364031149	19.04711729705067	9.95795269513754
H	11.40608278029741	19.55887326201524	8.56120664133476
H	6.31384679863862	16.94719831526930	9.30437907237781
H	7.32468902556313	19.16710176607651	8.81548745128215
H	7.50890075797988	13.51423153623395	7.47798165520284
H	6.17581927715798	14.36087851228152	8.30495928202191
H	6.75025727071215	14.92167141197277	6.71283076816170
H	16.01403549683224	19.03291542895190	6.75057696131608
H	16.55692740669294	17.36738236385966	6.99322623444000
H	17.60967635980815	18.52502352404069	6.13642749625740
H	12.11838661320837	18.10952681471259	1.08603331853481
H	13.17275642548472	19.47684401596714	0.65532083974140
H	13.84023238218668	17.83026767682047	0.77926792149757
H	12.44238858657475	20.98591953593616	2.72207557396837
H	11.28748379341024	19.74987066705120	3.29166441477693
H	12.46626340695146	20.43121508045919	4.41895636756933
H	17.02802215124896	19.66329889999724	3.71986490191508
H	15.18405885349082	20.50759561867992	2.31957033560466
H	17.11126474989528	14.61643971815463	3.14986061718148
H	18.00120594279675	16.67905720937015	4.17152987817252
H	13.37439246202914	14.50612003112375	1.49665704401852
H	14.70900792890220	15.58493490286797	1.05798844628026
H	15.03089084684387	13.86643253148362	1.39677555956733
H	14.62417339646874	13.63616825854345	5.30563295182594
H	13.36844613388526	13.24792696834501	4.12886731643421
H	15.05762854986019	12.76663521489848	3.80328445708511

I2, triplet

Fe	13.35480604871339	16.72177356939040	4.36940090544870
Fe	9.51393232604882	16.44409824771786	6.99563234708097
P	13.50163144449805	18.19298318347007	2.64712047642184
P	15.03669569566938	17.94823972867437	5.25366744791843
P	14.94825587380828	15.09141521058749	4.25413180063861
P	8.28942846823458	16.01925398101786	8.72484474520199
P	9.71691614898242	18.45027687620906	7.79347424471924
P	10.97597977941312	15.13811242173536	7.95150284609528
N	7.20711833904645	16.73941383611578	5.19480012811323
N	8.07677819150737	16.62501794483757	5.86408992932418
N	11.74401871230180	16.56477857978341	5.08190018831665
N	10.66242759247169	16.54320517134064	5.54858308942020
C	15.50417419829302	19.29889146886596	4.11754601291450
C	14.81606077004699	19.40796287716128	2.98090966440183
C	12.07862944600064	19.29924128716569	2.33707250082165
C	13.91031778517126	17.70420678200135	0.93009462639849
C	16.58216751929473	16.98333694005683	5.31993385012736
C	16.53222278750018	15.72366395411244	4.88799929742943

C	15.16238705259983	18.84229749303338	6.85359795590598
C	14.80213277849947	13.54419569058080	5.22448004281593
C	15.47307272797683	14.39689217672913	2.64223970722957
C	8.13006388609169	17.51743252293760	9.76240397673200
C	8.78049435803789	18.60690788299090	9.34809877784673
C	9.12159238090428	14.89153749055232	9.88021529573721
C	10.34619751088617	14.49758364957562	9.53054167338821
C	6.57539956010611	15.39431678229253	8.73114358425805
C	9.04148001374859	19.83290834788224	6.80800700516797
C	11.34759189155911	19.13866862694420	8.23660311566916
C	11.35226305521062	13.61200596764971	7.02869972527530
C	12.66346471306974	15.67080463018437	8.39536525525674
H	12.05311897704044	12.95352332897238	7.55736221048811
H	10.42227732626909	13.07142147297636	6.82882689997475
H	11.79086926691797	13.90615346916809	6.06785074366374
H	10.94217535038653	13.83439220263589	10.16475656292612
H	8.64739583386414	14.57305763447253	10.81303281349400
H	12.62538403300172	16.48777102851503	9.12273434615449
H	13.26518431677817	14.85295458140309	8.81152182851826
H	13.14375120500897	16.04225831096095	7.47939035079237
H	7.99698141534014	19.62731709872640	6.55068633415227
H	9.09851853956518	20.80031097833492	7.32233728490457
H	9.60680565217318	19.89550956243416	5.87061937368240
H	11.30706389987303	20.19420525117046	8.53238937806391
H	11.78613485785620	18.55908986783333	9.05485540186273
H	12.00543408363887	19.03198939985502	7.36524018589147
H	7.53961166757191	17.51586856018411	10.68392678879257
H	8.74859617801173	19.54043423625103	9.91875442893936
H	6.55399505194887	14.40240896033459	8.26700511840824
H	6.15235344182812	15.32474724241414	9.74056091892648
H	5.94356278247417	16.05971686290004	8.13238007218255
H	14.36669889146035	19.59375274604054	6.90827460647316
H	15.00556645903392	18.13151458097862	7.67289459305576
H	16.12800478783153	19.34367485426393	7.00049911344976
H	13.13077208803304	17.03326384936982	0.55109918164995
H	13.98865168673305	18.56320419801816	0.25196063709654
H	14.85937346860171	17.15826151274916	0.91591541915684
H	12.28803391483734	20.08275020771974	1.59725534137617
H	11.23209310170622	18.69571463145952	1.99007300307250
H	11.78045961901407	19.76475359758980	3.28225508091610
H	16.30157978849374	20.00590403539227	4.36773767171772
H	15.02951014155162	20.20637663910576	2.26216270011993
H	17.41643858425005	15.07779692660014	4.90998325751367
H	17.50881427413511	17.41358714576990	5.71258536918182
H	14.62545636483368	13.87981957779714	2.17783038456112
H	15.77792510055658	15.20898362200503	1.97436007761649
H	16.30411248674489	13.68680360675994	2.73680488563531
H	14.59154743793294	13.79515426996673	6.26947144469734

H	13.95322741293744	12.96229298586901	4.84709272135114
H	15.70494945605448	12.92091764138235	5.18058325345626

TS3, triplet, Freq=-84.40

Fe	13.55423114502809	16.53689540365617	4.10128387880715
Fe	9.19016328484688	16.00552602438909	7.20836863447205
P	13.46552325357726	18.49262160890483	2.97342078429586
P	15.04486891562264	17.59734948878810	5.42120688666315
P	15.28563748030795	15.12907746744940	3.65804215276336
P	8.18063438759393	16.34060100997040	9.09226093359873
P	9.59546067020876	18.12232204710778	7.13423614862776
P	10.76179326199230	14.99878390294904	8.36216432650304
N	7.06557637615538	15.73689502105132	5.22573267199463
N	7.86474952374542	15.82779342280283	5.98963172934704
N	11.94457412856663	15.99855036776073	4.80554333438326
N	10.92119689823228	15.58335574363200	5.03481833031737
C	15.34400832254578	19.27839820152696	4.77798584537680
C	14.63090495159773	19.67097539782494	3.72250079310095
C	11.93867317961327	19.49455246986632	2.91482817576284
C	13.98072143284861	18.56758080470657	1.21856996706571
C	16.69055305721551	16.83312055483818	5.23865681256861
C	16.78322704080572	15.73790852922399	4.48536521372047
C	15.08711651303954	17.95444968024731	7.22052096635509
C	15.22377817239177	13.39844573290866	4.23760646884059
C	15.89841801489487	14.89337006597120	1.95079636642871
C	8.24098690393843	18.12668151879110	9.48768818205918
C	8.86434615841349	18.91300543752957	8.60919956397562
C	9.18637606120259	15.67179432190195	10.46284340747686
C	10.33128562957317	15.07471176286005	10.12979046036844
C	6.49455522014271	15.93253882902789	9.68635049466510
C	8.84959534546608	19.13064451244488	5.79790568875068
C	11.26792131330753	18.87524800846100	7.16823981478964
C	10.98132579917604	13.19712721908800	8.11673697855197
C	12.51660308590795	15.51425851122273	8.41224526665224
H	11.67888977898237	12.74714246321940	8.83502346965194
H	10.00975201319754	12.69953496655864	8.20056825959317
H	11.35780698416737	13.02706064026772	7.10118148096585
H	10.99972338850383	14.65378823962064	10.88793544167147
H	8.87014995536678	15.75651558802798	11.50722085001853
H	12.58330387755343	16.55268725530603	8.75181123168891
H	13.12395685605858	14.88671686365408	9.07734079233938
H	12.93377679251395	15.46879625382810	7.39835121159447
H	7.78054575557103	18.90411429803127	5.72332793240049
H	8.97443840183742	20.21104272643262	5.94612010830477
H	9.31189729825300	18.84834441379402	4.84430741074637
H	11.24750706363671	19.97240201578042	7.13538956006811
H	11.78508972647125	18.56417582872496	8.08229681755638
H	11.84700792343897	18.50215290505844	6.31354958729363

H	7.78901024885114	18.53350345677216	10.39795472787765
H	8.95088820454031	19.99256480987307	8.77204571229191
H	6.35850993845111	14.84563809883708	9.65787265011846
H	6.29041186908681	16.28769457197647	10.70473993327843
H	5.75937929020487	16.37730204161968	9.00604818786124
H	14.19401462331371	18.52654491013386	7.49369555964263
H	15.06221016616264	17.01070837979815	7.77590788355339
H	15.97614173861722	18.52107546991572	7.52558619236796
H	13.28055523886160	17.98490740809029	0.60890222183858
H	14.00870867800547	19.59312361590888	0.83015306448300
H	14.97521892780895	18.12268596928251	1.10755514482159
H	12.08269320787028	20.46971821031208	2.43216023420588
H	11.16590447870217	18.93985107846830	2.37038509627878
H	11.57384692768257	19.65206654176567	3.93524445461882
H	16.06093363600936	19.95189023975732	5.25783624979578
H	14.74202435714061	20.67671547229731	3.30386200105216
H	17.73517294468936	15.21267699128969	4.35593107697125
H	17.56567338453453	17.24036141871856	5.75433394833349
H	15.12106392142306	14.40806922107032	1.34959948741113
H	16.11457920951553	15.86652245218762	1.49753605507549
H	16.80526917691633	14.27729333836431	1.91004835570331
H	14.96823616918620	13.38688686200718	5.30212242842319
H	14.42987227190561	12.86734056109497	3.70018210950897
H	16.17116004699761	12.86488135523426	4.08850279230440

2m, singlet

Fe	11.31097973483197	16.29329819152405	8.07552880793831
Fe	13.67933172698809	16.85311419588423	4.34904625840322
P	11.42131004189851	16.66478988739435	10.18401756324360
P	9.45009471788835	17.03740496090316	7.42021688611722
P	10.33189835506977	14.50532721330875	8.57325836003432
P	13.33613182739897	15.91278324763987	2.46611009418088
P	15.69359594526816	17.03113379299666	3.72950108263316
P	13.50909633476074	18.93247309510684	4.06205407226568
N	12.40023220627115	16.40588086885568	6.86126614251779
N	12.99140399314665	16.46185270048707	5.78057516381457
C	10.73859918913105	18.11628231655210	11.06700520970112
C	13.10799423457627	16.64151243086826	10.88978773488285
C	8.46311677099763	18.39118494144798	8.16067066729232
C	9.42405251692278	17.54292396339434	5.66773648611817
C	10.88805785049836	12.85380291541913	8.04446989090642
C	10.63295722133045	15.29838773357575	11.10711313458913
C	10.13489154521188	14.30115698673071	10.37426788669086
C	8.21266589555392	15.69521323598235	7.45492472828543
C	8.60391029263831	14.54053726607668	7.99535198629862
C	12.23024010733263	16.54255136037356	1.14961171077678
C	12.76308133876525	14.17944539776368	2.55672098083611
C	17.13962246582859	16.47737594515963	4.69136434466527

C	14.89559875649648	15.73532791079497	1.53179853631439
C	15.98313822714810	16.25138227766994	2.10603700457658
C	12.35113328527593	19.77550281200497	2.92404540137194
C	13.18904090918028	19.85311575105004	5.60345503862095
C	15.10521018376711	19.63940219108778	3.53200509906226
C	16.10933700590792	18.77398422112693	3.38371946921187
H	12.73136907601700	13.68532494328247	1.57723822234222
H	11.75974017012106	14.16428586053329	2.99747856963178
H	13.42441885607869	13.62000617042010	3.22573449871254
H	12.17890520845021	15.86820865637957	0.28553193219406
H	12.57915992590444	17.52169534515084	0.80602240259813
H	11.22035266112034	16.66788795628749	1.55660175823032
H	16.96878131620009	16.20997613906571	1.63365142630328
H	14.93424149460601	15.24661973922985	0.55316353393913
H	15.23394068236834	20.71086303082382	3.35010542214841
H	17.11104309447536	19.09040529367363	3.07852612961436
H	13.96451276126797	19.59700306106229	6.33220737149242
H	12.23698418795217	19.51217940678649	6.02729214715047
H	13.16075483761972	20.94076166948222	5.46243747227958
H	12.54649565555981	19.47000700690630	1.89147039843566
H	12.42573429643942	20.86845867721211	2.98616108647830
H	11.32552736803532	19.47982350798529	3.17360569698777
H	18.09341366326302	16.69480248590230	4.19485654822409
H	17.06217574141727	15.39807551915696	4.86056788085299
H	17.12350249629357	16.97103297063311	5.66885633854321
H	10.95414540680612	12.83956768565543	6.95132519939955
H	10.22088771976913	12.04906595758772	8.37715361383547
H	11.89257347963540	12.67397586730223	8.44172386100593
H	7.19631592698451	15.84073872417422	7.07537883424272
H	7.93389852608426	13.68042486264014	8.08279267852119
H	9.06362326900495	19.30787215373019	8.18246234987775
H	8.19875955044615	18.13558572267414	9.19215342486864
H	7.53904870909284	18.59070425658640	7.60333873634718
H	9.97499935018477	18.48550666177114	5.56792484260585
H	8.40888542062637	17.68311151994160	5.27574920445962
H	9.95891671137001	16.79711075130301	5.06995615723677
H	11.20690902776214	19.02523660064206	10.67213387436902
H	10.91764035337851	18.07253797663824	12.14875911985120
H	9.66084919853741	18.19306493995697	10.89312925259107
H	9.64980543953662	13.43076263952333	10.82515589374498
H	10.57552003892568	15.29627005074817	12.20024632179812
H	13.66305061115379	17.50283238018096	10.50089952377471
H	13.62434594228090	15.73774213138536	10.55138565156008
H	13.11620914514233	16.67690386640084	11.98666288240009
